# Dynamic circulating tumor DNA profiling of ESR1 mutations in HR+/HER2− advanced breast cancer: from prognostic biomarker to clinical decision-making – a comprehensive review

**DOI:** 10.3389/fonc.2026.1881097

**Published:** 2026-07-31

**Authors:** Saiyu Ren, Pengwei Li, Dongcheng Zhu, Lei Zhao, Xiliang Zhang

**Affiliations:** 1 Department of General Surgery, The Sixth Medical Center of Chinese PLA General Hospital, Beijing, China; 2 Department of Hepatobiliary Surgery, The Sixth Medical Center of Chinese PLA General Hospital, Beijing, China; 3 Department of Radiology, The Sixth Medical Center of Chinese PLA General Hospital, Beijing, China

**Keywords:** breast cancer, ESR1 mutation, circulating tumor DNA, CDK4/6 inhibitor, endocrine therapy, precision medicine

## Abstract

**Background:**

The combination of CDK4/6 inhibitors (CDK4/6i) and endocrine therapy (ET) is the standard first−line regimen for hormone receptor-positive, human epidermal growth factor receptor 2−negative (HR+/HER2-) metastatic breast cancer (MBC). Resistance remains a challenge, with estrogen receptor 1 gene (*ESR1*) mutations serving as a central mechanism. Liquid biopsy enables non-invasive monitoring via circulating tumor DNA (ctDNA), with *ESR1* mutations increasingly recognized as dynamic molecular events rather than static markers.

**Objective:**

This comprehensive review evaluates the prognostic and predictive value of baseline and on-treatment ctDNA *ESR1* mutations in HR+/HER2- advanced breast cancer patients receiving CDK4/6i and ET, and explores their evolution into dynamic intervention nodes for precision decision-making.

**Methods:**

Following PRISMA guidelines, we searched major databases and conference proceedings up to February 2026. Studies reporting ctDNA *ESR1* mutations and clinical outcomes in CDK4/6i plus ET-treated patients were included. Due to substantial clinical and methodological heterogeneity, a quantitative meta−analysis was not feasible; instead, a narrative synthesis was performed.

**Results:**

Nine high-quality studies met eligibility criteria. Key findings: (1) Baseline *ESR1* mutations, particularly Y537S, are adverse prognostic factors; high ctDNA tumor fraction (≥10%) identifies patients deriving greater benefit from first-line CDK4/6i (PFS2 HR 0.58). (2) ctDNA clearance by cycle 2 day 1 predicts prolonged PFS (HR = 0.44). (3) Pre-emptive switching to camizestrant upon ctDNA-detected *ESR1* mutations reduced progression risk by 56% (HR = 0.44, P<0.0001) in the SERENA-6 trial.

**Conclusions:**

Baseline ctDNA *ESR1* mutations define a more aggressive disease subtype and have evolved from molecular drivers to actionable clinical decision nodes. However, routine clinical adoption requires prospective validation, harmonized testing standards, and consensus on monitoring protocols. This review provides a comprehensive evidence-graded framework to guide future research and clinical implementation.

## 1 Introduction

Breast cancer ranks first in incidence among female malignancies worldwide (1). Substantial improvements in early detection and systemic therapy have markedly enhanced the prognosis for patients with breast cancer (2). However, hormone receptor-positive, human epidermal growth factor receptor 2-negative (HR+/HER2-) disease, which accounts for approximately 65–70% of all breast cancers, remains the predominant phenotype in the advanced setting and continues to confront clinicians with significant therapeutic challenges (3). The advent of cyclin-dependent kinase 4/6 inhibitors (CDK4/6i) in combination with endocrine therapy (ET) has established a new standard of care in the first-line treatment of HR+/HER2- metastatic breast cancer (MBC), conferring significant and reproducible improvements in both progression-free survival (PFS) and overall survival (OS) (4, 5). As the combination of CDK4/6i and endocrine therapy (ET) becomes widely adopted as the first-line treatment for HR+/HER2- advanced breast cancer, resistance remains an inevitable challenge. Previous studies have shown that approximately 20% of patients exhibit primary resistance to CDK4/6i plus ET, while nearly all patients eventually experience disease progression. Moreover, therapeutic options following progression remain limited, with substantially attenuated efficacy (6–8). Elucidating the mechanisms underlying resistance and developing clinically validated biomarkers to guide treatment decisions have therefore emerged as critical priorities for optimizing precision oncology in this setting, with *ESR1* mutations and ctDNA monitoring emerging as key focuses of research.

Emerging evidence suggests that these resistance phenotypes may be driven, at least in part, by the same genetic alterations that confer endocrine therapy resistance. Mutations in the estrogen receptor 1 (*ESR1*) gene, which encodes estrogen receptor α (ERα), represent one of the most prevalent mechanisms of acquired resistance to endocrine therapy in HR+ breast cancer (9). The critical role of *ESR1* mutations in the context of CDK4/6i resistance is underscored by recent real-world and preclinical evidence: a large analysis of patients with HR+ metastatic breast cancer (n=3,958) demonstrated that *ESR1* mutations—particularly Y537S and D538G—were significantly enriched following CDK4/6i exposure, with preclinical models confirming that these mutations directly confer resistance to palbociclib (10). These mutations, predominantly clustered within the ligand-binding domain (LBD), confer ligand-independent constitutive activation of ERα by stabilizing its transcriptionally active conformation, thereby rendering aromatase inhibitors (AIs) ineffective (10). Among patients progressing on prior AI therapy, *ESR1* mutations are detectable in 30%–40% of cases, (11). and multiple studies have confirmed that patients with these mutations have significantly shorter progression-free survival (PFS) and overall survival (OS) compared to those with wild-type status (12–14). Thus, identifying *ESR1* mutation status not only aids in determining patient prognosis but also provides crucial evidence for the selection of subsequent precision treatment strategies, particularly in the context of CDK4/6i resistance.

The emergence of liquid biopsy has fundamentally reshaped the diagnostic and therapeutic landscape for HR+/HER2- metastatic breast cancer. Circulating tumor DNA (ctDNA)-based *ESR1* mutation detection has advanced from technical validation to high-level clinical evidence, offering several distinct advantages over traditional tissue biopsy (15, 16). It is minimally invasive (requiring only a blood draw), highly sensitive, capable of overcoming tumor spatial heterogeneity, and uniquely enables dynamic monitoring—a feature particularly valuable for tracking treatment-emergent mutations (17, 18). While routine testing is not recommended in early-stage disease due to low mutation rates (~1%), it is clinically relevant in advanced disease—particularly following endocrine therapy—as *ESR1* mutation prevalence increases with treatment duration and accumulated lines of therapy. Beyond static assessment, longitudinal ctDNA monitoring during treatment has emerged as a promising strategy, with preliminary evidence suggesting that early intervention based on emergent *ESR1* mutations, even before radiographic progression, may confer survival benefits. This notion is reinforced by real-world data from Chaudhary et al., who analyzed 5,910 patients and found that exposure to CDK4/6i plus AI leads to a significantly higher incidence of *ESR1* mutations—independent of treatment duration—and is associated with increased co-occurring mutations in multiple oncogenic pathways (19). Notably, ctDNA can detect *ESR1* mutations a median of 7.8 months prior to radiographic progression, providing a critical window for early intervention.

Collectively, these findings underscore a fundamental paradigm shift: *ESR1* mutations have evolved from static prognostic markers to dynamic, potentially actionable clinical decision nodes, highlighting the urgent need for treatment strategies tailored to the continuously evolving tumor genomic landscape. This paradigm has now been prospectively validated. The SERENA-6 trial demonstrated that pre-emptive switching to camizestrant plus CDK4/6i, guided by ctDNA-detected emergent *ESR1* mutations, significantly reduced the risk of deterioration in pain, dyspnea, and emotional functioning (HR 0.51–0.57) with favorable tolerability, providing the highest level of evidence for *ESR1* mutation-driven precision therapeutic switching (20, 21).

Against this backdrop, this comprehensive review aims to: (1) critically evaluate the prognostic and predictive value of baseline and on-treatment ctDNA *ESR1* mutations in the context of CDK4/6i-based therapy; (2) synthesize emerging evidence on early ctDNA kinetics as a molecular readout of therapeutic efficacy; (3) delineate the functional and clinical heterogeneity among *ESR1* mutation subtypes; and (4) assess the translational impact of recently established intervention paradigms—most notably SERENA-6—that have redefined “molecular relapse” as an actionable trigger for therapeutic switching. By integrating these evidence streams, we propose a dynamic precision management framework that converges baseline risk stratification, real-time efficacy monitoring, and clonal evolution tracking—aiming to advance the management of HR+/HER2- advanced breast cancer from static risk assessment toward dynamic, precision-guided intervention. This review fills the existing research gap by systematically integrating evidence on *ESR1* mutation subtypes, ctDNA dynamics, and intervention paradigms, providing a comprehensive basis for clinical practice and future research.

## 2 Biology and detection science of *ESR1* mutations

### 2.1 Molecular structure and clinical significance

In HR+/HER2- breast cancer, *ESR1* mutations are the most common acquired mechanism of endocrine therapy resistance (22). The major mutation hotspots are concentrated in the ligand-binding domain (LBD), including D538, Y537, L536, P535H, and V534E (23–26). Missense mutations—most commonly Y537S and D538G—stabilize the transcriptionally active conformation of ERα, enabling ligand-independent activation of downstream oncogenic signals (27, 28). While most patients develop single point mutations, approximately 30% exhibit co-mutations at multiple *ESR1* sites, a pattern associated with stronger drug resistance and poorer prognosis (29).

Patients harboring *ESR1* mutations experience significantly shorter PFS when rechallenged with CDK4/6i or switched to other targeted therapies, suggesting limited response to subsequent endocrine-targeted therapies (30, 31). Y537S and D538G are significantly enriched in CDK4/6i-treated patients and actively drive resistance (10). Structural studies show Y537S exhibits 2–3 times higher transcriptional activity than D538G, translating into shorter survival and lower response rates to conventional endocrine therapy (32, 33). Detecting mutation subtypes informs prognostic assessment and treatment intensity, supporting integration of subtyping into routine testing (34, 35).

Among patients progressing on aromatase inhibitor (AI) therapy, *ESR1* mutation detection rates reach 40%–50%; after one year of first-line CDK4/6i plus AI, cumulative incidence reaches 50% (36, 37). However, *ESR1*-mutant breast cancer maintains sensitivity to selective estrogen receptor downregulators (SERDs) such as fulvestrant and novel oral SERDs. The PADA-1 trial showed that switching from AI to fulvestrant upon ctDNA-detected *ESR1* mutation emergence prolongs PFS (38). The EMERALD study demonstrated that in CDK4/6i-pretreated populations, elacestrant extended median PFS from 1.9 to 3.8 months (45% risk reduction), leading to FDA approval as the first oral SERD for *ESR1*-mutant advanced breast cancer (39). Thus, *ESR1* mutation detection serves as a molecular trigger for treatment class switching rather than a mere declaration of resistance.

### 2.2 Detection technologies for *ESR1* mutations in ctDNA

ctDNA testing has become the primary means of *ESR1* mutation monitoring. Mainstream technologies include digital PCR (ddPCR) and next-generation sequencing (NGS). ddPCR offers ultra-high sensitivity (allele fraction as low as 0.01–0.1%) for quantitative tracking of known hotspot mutations and has been successfully used in trials such as PADA-1, where it enabled rapid, cost-effective screening of D538G and Y537S mutations. However, ddPCR is limited to predefined targets and cannot detect novel or rare mutations outside the selected hotspots. NGS enables unbiased scanning of the entire *ESR1* LBD and simultaneous analysis of co-mutation profiles (e.g., *PIK3CA*, *TP53*) but typically has lower sensitivity (variant allele frequency [VAF] detection limit ~0.5–1%) and higher cost (22, 37). Importantly, there is currently no consensus on optimal VAF thresholds for defining “positive” *ESR1* mutation status; thresholds used in pivotal trials ranged from 0.1% (ddPCR-based studies) to 1% (NGS-based studies). This heterogeneity directly impacts inter-study comparability and clinical interpretation. Furthermore, pre-analytical factors—including blood collection tube type (e.g., Streck vs. EDTA), time from draw to plasma separation, and centrifugation protocols—significantly affect ctDNA yield and integrity, yet standardized workflows are lacking across institutions. These technical challenges represent major barriers to widespread clinical adoption and highlight the urgent need for harmonized guidelines.

Clinical integration spans multiple contexts. Regular monitoring enables early detection of emergent mutations before radiographic progression, as validated in SERENA-6 where ctDNA identified *ESR1* mutations a median of 7.8 months prior to imaging progression (20, 21). Early ctDNA kinetics serve as efficacy surrogates: the BioItaLEE trial demonstrated that clearance by cycle 2 day 1 correlates with significantly prolonged PFS (HR = 0.44) (40). In the post-progression setting, comprehensive NGS profiling of *ESR1* and co-occurring mutations (e.g., *PIK3CA*, *TP53*) informs subsequent treatment selection, as exemplified by the EMERALD trial. This multi-setting framework positions ctDNA *ESR1* testing as a continuous surveillance system that captures the evolving genomic landscape under therapeutic pressure (41). Future efforts should focus on establishing unified testing standards and quality control systems to promote routine application, including standardized monitoring intervals and intervention thresholds.

## 3 Methods

### 3.1 Study selection

This comprehensive review was conducted in accordance with PRISMA 2020 guidelines. Although not registered in PROSPERO (due to the narrative synthesis design and the rapidly evolving nature of the evidence base), the search strategy and selection process strictly followed PRISMA standards. We searched PubMed, Embase, Web of Science, Cochrane Library, and conference proceedings (ASCO, ESMO, SABCS) from inception to February 2026. The search strategy combined terms related to “breast cancer”, “HR+/HER2-”, “*ESR1* mutation”, “ctDNA”, “CDK4/6 inhibitor”, and “endocrine therapy”. Two independent reviewers conducted screening and data extraction; discrepancies were resolved by a third reviewer.

Inclusion criteria: (1) Patients with HR+/HER2- advanced breast cancer; (2) ctDNA-based *ESR1* mutation detection; (3) prognostic or predictive value in CDK4/6i plus ET-treated patients; (4) reporting of clinical outcomes (PFS, OS, ORR, or HR). Exclusion criteria: (1) Case reports, reviews, letters; (2) lacking ctDNA *ESR1* data or clinical outcomes; (3) duplicate cohorts.

**Figure 1:**
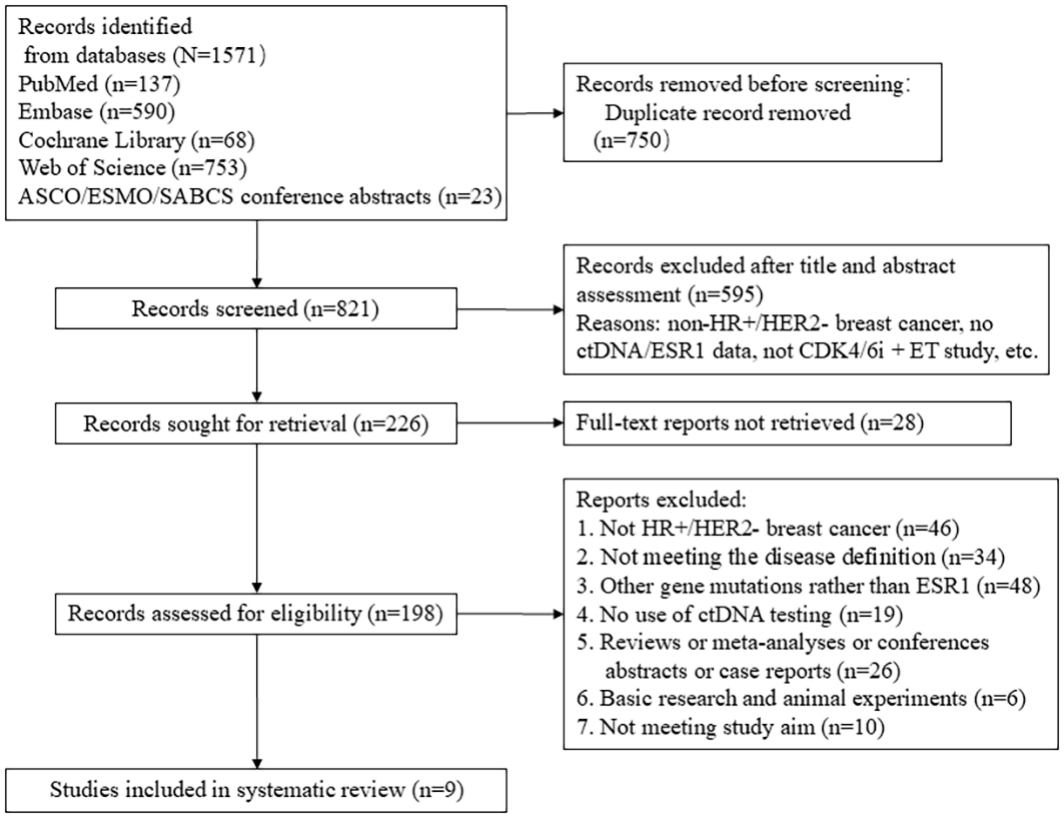
PRISMA flow diagram of study selection process. PRISMA 2020 flow diagram showing study selection process: 1571 records identified, 750 duplicates removed, 821 records screened, 595 excluded by title/abstract, 226 full-text articles assessed, 9 studies included in comprehensive review.

A total of 1,571 records were identified. After removing 750 duplicates, 821 records were screened. Following title/abstract assessment, 595 records were excluded, leaving 226 full-text reports assessed. Ultimately, 9 studies met all criteria and were included (**Figure 1**).

### 3.2 Data extraction

Two reviewers extracted data using a standardized form, including study characteristics, patient population, biomarker methodology, genomic features, clinical outcomes, dynamic monitoring data, and intervention details.

### 3.3 Quality assessment

Methodological quality was assessed independently by two reviewers. RCTs were assessed using Cochrane RoB 2.0 tool; key trials (SERENA-6, PADA-1, EMERALD) were rated low risk of bias. Observational studies were assessed using Newcastle–Ottawa Scale; all scored 7–9 stars, indicating high quality (**Table 1**). The detailed Newcastle-Ottawa Scale scoring is presented (**Table 2**).

**Table 1A:** Risk of bias and quality assessment of included studies.

Study	Random-ization process	Deviations from interventions	Missing outcome data	Outcome measurement	Selective reporting	Overall risk of bias
MONARCH 3	Low	Low	Low	Low	Low	Low
PALOMA-3	Low	Low	Low	Low	Low	Low
SONIA	Low	Some concerns¹	Low	Low	Low	Some concerns
PADA-1	Low	Low	Low	Low	Low	Low
SERENA-6	Low	Low	Low	Low	Low	Low
EMERALD	Low	Low	Low	Low	Low	Low
postMONARCH	Low	Low	Low	Low	Low	Low
MAINTAIN	Low	Low	Low	Low	Low	Low

SONIA was rated as having some concerns in the “deviations from interventions” domain due to its open-label design. However, the ctDNA translational analysis included in this review was pre-specified, centrally conducted, and assessed independently of treatment assignment, mitigating bias for the findings incorporated in this comprehensive review.

(A) Risk of bias assessment for randomized controlled trials (Cochrane RoB 2.0).

**Table 1B:** Quality assessment for non-randomized study (Newcastle–Ottawa Scale)

Study	Selection (0–4 ★)	Comparability (0–2 ★)	Outcome (0–3 ★)	Total score (0–9)	Quality level
BioItaLEE	★★★★	★★	★★★	9	High

### 3.4 Heterogeneity assessment

Due to substantial clinical and methodological heterogeneity across studies (including differences in CDK4/6i agents, endocrine therapy backbones, ctDNA detection platforms, VAF thresholds, and patient populations), quantitative meta-analysis was not feasible. A narrative synthesis was performed, stratifying by study design, patient population, and biomarker methodology. All interpretations are clearly distinguished from the available evidence, and conclusions are limited to the current level of evidence.

## 4 Systematic evaluation of clinical evidence

We systematically reviewed and compared key studies (**Table 3** and **Supplementary Table 1**). The evidence hierarchy progresses from establishing baseline *ESR1* mutations as adverse prognostic factors (MONARCH 3, PALOMA-3), through refining treatment timing (SONIA) and early efficacy readouts (BioItaLEE, PADA-1), to the pivotal validation of ctDNA-detected *ESR1* mutations as actionable intervention triggers (SERENA-6). Complementary trials in the post-CDK4/6i setting (EMERALD, postMONARCH, MAINTAIN) further establish therapeutic implications across the disease continuum.

**Table 2:** Detailed NOS component scoring for BioItaLEE.

Component	Score	Justification
Selection (4 stars)		
Adequate case definition	★	Prospective enrollment, predefined inclusion criteria (HR+/HER2- advanced breast cancer, first-line ribociclib + letrozole)
Representativeness of cases	★	Consecutive enrollment from multicenter Italian trial (n=287)
Selection of controls	★	Internal control group (patients without baseline ctDNA mutations) from same cohort
Definition of controls	★	Control group clearly defined with identical follow-up protocol
Comparability (2 stars)		
Control for important factors	★★	Multivariable Cox regression adjusting for clinicopathological factors; stratification by ESR1 mutation status
Outcome (3 stars)		
Outcome assessment	★	PFS assessed with predefined criteria; ctDNA analysis performed blinded to clinical outcomes
Adequate follow-up length	★	Median follow-up 23.4 months, sufficient to capture PFS endpoint
Adequacy of follow-up	★	ctDNA samples available for >80% of enrolled patients at each timepoint (baseline: 263/287, C1D15: 238/287, C2D1: 241/287)

**Table 3:** Key clinical trials informing the evolution of ctDNA *ESR1* mutations from prognostic biomarkers to dynamic intervention targets in HR+/HER2− advanced breast cancer.

Study	Study design	Patient population/line of therapy	Sample size (total/ctDNA subgroup)	ctDNA detection method	ESR1 mutation subtype analysis	Key findings	Reference
MONARCH 3	Randomized, double-blind, phase III	First-line	493/295	NGS	Yes	Baseline ESR1 mutations were adverse prognostic factors. Final OS analysis (8.1 years): median OS 66.8 vs. 53.7 months (HR = 0.804; 95% CI 0.637-1.015; P = 0.0664). In visceral metastasis subgroup, median OS 63.7 vs. 48.8 months (HR = 0.758; 95% CI 0.558-1.030; P = 0.0757).	(42), Ann Oncol
PALOMA-3	Randomized, double-blind, phase III	Second-line or later (post-endocrine therapy progression)	521/395	ddPCR (PIK3CA) + NGS (ESR1/TP53)	Yes	PFS: median 9.5 vs. 4.6 months (HR = 0.46). OS update (73.3 months): median 34.8 vs. 28.0 months (HR = 0.81; 95% CI 0.65-0.99). ctDNA analysis showed consistent OS benefit regardless of ESR1, PIK3CA, or TP53 mutation status.	Lancet Oncol; (43), CCR
SONIA	Randomized, open-label, phase III (translational analysis)	First-line vs. second-line (timing of CDK4/6i use)	1050/1013	mFast-SeqS (aneuploidy score)	No	Primary OS: first-line vs. second-line CDK4/6i, median 47.9 vs. 48.1 months (HR = 0.91; 95% CI 0.77-1.07; P = 0.24). Premenopausal subgroup OS benefit (HR = 0.53). ctDNA analysis: high baseline ctDNA tumor fraction (≥10%) identified patients deriving greater PFS2 benefit from first-line CDK4/6i (HR = 0.58).	(44) JAMA Oncol
BioItaLEE	Prospective, phase III translational study	First-line (ribociclib + letrozole)	287/263 (baseline), 238 (C1D15), 241 (C2D1)	NGS (39-gene panel)	Yes	Median PFS 23.4 months. No baseline mutations: better prognosis (HR = 0.41). Among baseline mutation-positive patients, ctDNA clearance at C2D1 associated with reduced progression risk (HR = 0.44). Among baseline mutation-negative patients, 22.7% developed detectable mutations during treatment.	(40), CCR
PADA-1	Randomized, open-label, phase III (dynamic intervention + translational analysis)	First-line (rising ctDNA ESR1 during AI + palbociclib, no radiographic progression)	1017/172 (randomized)	ddPCR (ESR1 screening)	Yes	Early switching to fulvestrant + palbociclib vs. continuation of AI + palbociclib: PFS HR = 0.61 (95% CI 0.43-0.86; P = 0.004), median PFS 11.9 vs. 5.7 months.	(11), Lancet Oncol
SERENA-6	Randomized, double-blind, phase III (dynamic intervention)	First-line (emergent ctDNA ESR1 after ≥6 months of AI + CDK4/6i, no radiographic progression)	3,256 (screened)/315 (randomized)	High-sensitivity NGS (monitoring every 2–3 months)	Yes (Y537S/D538G)	Early switching to camizestrant + CDK4/6i vs. continuation of AI + CDK4/6i: PFS HR = 0.44 (95% CI 0.31-0.60; P<0.0001), median PFS 16.0 vs. 9.2 months; time to QoL deterioration HR = 0.54; discontinuation rates 1.3% vs. 1.9%.	(20), NEJM
EMERALD	Randomized, open-label, phase III	Previously treated (progression on prior CDK4/6i, 1–2 lines of ET, ≤1 line of chemotherapy)	477/228 (ESR1-mutated)	ctDNA-based ESR1 detection	Yes (Y537S/N, D538G)	In ESR1-mutated population, elacestrant vs. standard of care: PFS HR = 0.55 (95% CI 0.39-0.77). Prior CDK4/6i ≥12 months subgroup: HR = 0.41 (95% CI 0.26-0.63). Both Y537S/N and D538G mutations derived comparable benefit (median PFS 9.0 vs. 1.9 months).	(20), CCR
postMONARCH	Randomized, double-blind, phase III	Previously treated (progression on prior CDK4/6i, or relapse during/after adjuvant CDK4/6i)	368/320	NGS	Yes	Abemaciclib + fulvestrant vs. fulvestrant: PFS HR = 0.73 (95% CI 0.57-0.95; P = 0.017); by BICR: HR = 0.55 (95% CI 0.39-0.77). ESR1 mutation detection rate 40-51%. ESR1-mutated: HR = 0.79; non-mutated: HR = 0.78.	(33), JCO
MAINTAIN	Randomized, double-blind, phase II	Previously treated (post-CDK4/6i progression)	120/84	NGS	Yes	Ribociclib + endocrine therapy vs. placebo: PFS HR = 0.57 (95% CI 0.39-0.95).	(30), JCO

### 4.1 Prognostic value analysis

Pooled evidence indicates that baseline *ESR1* mutations are associated with shorter PFS in both first-line and second-line settings, with hazard ratios typically ranging from 2.0 to 3.0. The magnitude of this prognostic effect varies across studies, a heterogeneity that may be attributed to differences in patient populations and treatment contexts.

In second-line studies (e.g., PALOMA-3), where patients generally have a poorer prognosis and a higher frequency of *ESR1* mutations, the observed “prognostic effect” may be partially diluted. Notably, the final overall survival (OS) analysis of the PALOMA-3 study, after a long-term follow-up of 73.5 months, demonstrated that palbociclib plus fulvestrant significantly improved OS compared with placebo plus fulvestrant (median OS, 34.8 vs. 28.0 months; HR, 0.81; 95% CI, 0.65-0.99) (43). Exploratory ctDNA analysis further revealed a consistent OS benefit from palbociclib treatment, regardless of *ESR1*, *PIK3CA*, or *TP53* mutation status. This suggests that although *ESR1* mutations serve as adverse prognostic factors, they do not diminish the relative benefit derived from the combination of a CDK4/6 inhibitor and endocrine therapy.

Conversely, in first-line studies (e.g., MONARCH 3), baseline *ESR1* mutations may signify a biological subtype with greater intrinsic aggressiveness or primary resistance to aromatase inhibitors (AIs), thereby exerting a more pronounced impact on prognosis. Final overall survival analysis of MONARCH 3, with a median follow-up of 8.1 years, demonstrated that among patients with baseline ctDNA *ESR1* mutations, median OS was 66.8 months in the abemaciclib plus NSAI arm versus 53.7 months in the placebo plus NSAI arm (HR = 0.804; 95% CI 0.637-1.015; P = 0.0664). In the visceral metastasis subgroup, median OS was 63.7 months versus 48.8 months (HR = 0.758; 95% CI 0.558-1.030; P = 0.0757). A dedicated ctDNA translational analysis of MONARCH 3 demonstrated that baseline *ESR1* mutations were detected in only 5% of patients, and although associated with numerically shorter PFS in both arms, the benefit of adding abemaciclib to NSAI was preserved regardless of *ESR1* mutation status (interaction P = 0.163) (45). Furthermore, longitudinal ctDNA analysis revealed that acquired *ESR1* mutations at the end of treatment were significantly less frequent in patients receiving abemaciclib plus NSAI compared with placebo plus NSAI (19.8% vs. 31.6%), suggesting that abemaciclib may delay the emergence of *ESR1*-mediated resistance (42). Subgroup analysis further revealed that the prognostic value of baseline *ESR1* mutations is more significant in patients with a high baseline ctDNA tumor fraction (aneuploidy score ≥10%), underscoring the need for an integrated risk assessment that combines mutation status with quantitative ctDNA indicators.

Treatment after progression on first-line CDK4/6i is a critical clinical question. The phase III postMONARCH trial confirmed that abemaciclib plus fulvestrant significantly improved progression-free survival in patients (HR 0.73; 95% CI 0.57-0.95; P = 0.017; by blinded independent central review (BICR): HR 0.55; 95% CI 0.39-0.77). Exploratory ctDNA analysis showed consistent PFS benefit regardless of *ESR1* or *PIK3CA* mutation status (*ESR1* mutated: HR 0.79; non-mutated: HR 0.78). As the first phase III study to demonstrate benefit from continued CDK4/6i combined with a switch in endocrine therapy in a CDK4/6i-pretreated population, postMONARCH provides a treatment backbone and control benchmark for *ESR1* mutation-guided pre-emptive intervention strategies. These two approaches complement each other, collectively advancing the paradigm shift in HR+/HER2- advanced breast cancer from static risk stratification towards dynamic precision intervention.

### 4.2 Predictive value and treatment effect modification

Current evidence more strongly supports the prognostic value of *ESR1* mutations rather than their predictive value in the context of CDK4/6i plus endocrine therapy. In other words, while patients harboring *ESR1* mutations generally have a poorer overall prognosis, existing data have not consistently demonstrated that their relative benefit from CDK4/6i treatment (compared to placebo plus endocrine therapy) is significantly inferior to that of *ESR1* wild-type patients. For example, in the analysis by Goetz et al., although the *ESR1*-mutant subgroup exhibited shorter absolute PFS, CDK4/6i still showed a trend toward treatment benefit within this subgroup, suggesting that mutation status does not completely negate the efficacy of CDK4/6i. However, this trend varies by mutation subtype: Y537S-mutant patients derive less benefit from CDK4/6i monotherapy compared to D538G-mutant patients, indicating the need for subtype-specific treatment adjustments.

However, under specific clinical contexts, *ESR1* mutation status demonstrates clear predictive value for subsequent treatment selection. For patients who develop *ESR1* mutations during aromatase inhibitor therapy, switching to fulvestrant or novel oral SERDs has been proven to significantly improve prognosis—serving as a quintessential example of the evolution of *ESR1* mutations from a “prognostic marker” to a “predictive marker.” (41). The EMERALD trial provided the highest-level evidence to date, demonstrating that in patients with *ESR1*-mutant advanced breast cancer who had progressed on prior CDK4/6i, elacestrant reduced the risk of progression by 45% compared to standard endocrine therapy (HR = 0.55; 95% CI 0.39-0.77), with a particularly pronounced benefit in patients who had received CDK4/6i for ≥12 months (HR = 0.41; 95% CI 0.26-0.63). In subgroup analyses by mutation subtype, both Y537S/N and D538G mutations derived comparable benefit, with median PFS of 9.0 months versus 1.9 months in the control arm. Notably, the predictive value of *ESR1* mutations is enhanced when combined with co-mutation analysis, such as *TP53* or *PIK3CA* mutations, which further refine treatment response prediction.

### 4.3 Impact of co-mutations and tumor heterogeneity

*ESR1* mutations do not exist in isolation. Studies have shown that they frequently co-occur with mutations in other genes such as *TP53* and *PIK3CA*, forming complex co-mutation landscapes. Research by O’Leary et al. found that patients with co-existing *ESR1* and *TP53* mutations have an extremely poor prognosis, suggesting that co-mutation analysis can further refine risk stratification (38). In MONARCH 3, comprehensive ctDNA profiling revealed that 81% of patients harbored at least one baseline genomic alteration, with *PIK3CA* (37.6%) and *TP53* (25.4%) being the most frequent (45). Notably, acquired alterations in RB1 and MYC were observed exclusively in the abemaciclib arm (5.3% and 4.6%, respectively), potentially representing novel mechanisms of acquired resistance to CDK4/6 inhibition (42). These findings underscore the value of longitudinal ctDNA monitoring in capturing emerging resistance mechanisms that may not be present at baseline.

Furthermore, *ESR1*-mutant clones may represent only a subset of the tumor cell population. Their competition and evolution with wild-type clones, as well as their dynamic changes under therapeutic pressure, collectively determine the eventual resistance phenotype. This understanding underscores the necessity of comprehensive genomic profiling and dynamic monitoring—detecting a single mutation at a single time point is insufficient to capture the full picture of tumor evolution. Tumor spatial and temporal heterogeneity highlights the limitations of single-time-point tissue biopsy, further emphasizing the value of longitudinal ctDNA monitoring in capturing clonal evolution.

For instance, the BioItaLEE trial demonstrated that co-occurring *TP53* or MYC mutations were associated with early progression on ribociclib plus letrozole (40). In this study, among patients with detectable baseline ctDNA mutations, those achieving ctDNA clearance at cycle 2 day 1 had a significantly reduced risk of progression (HR = 0.44), whereas persistent co-mutations indicated primary resistance. Similarly, the PADA-1 translational analysis revealed that the number of persistent driver mutations during early treatment was strongly associated with inferior overall survival (HR = 1.51). These findings underscore the need for comprehensive genomic profiling beyond isolated *ESR1* assessment (46).

### 4.4 From static biomarker to dynamic interventional target

**Figure 2:**
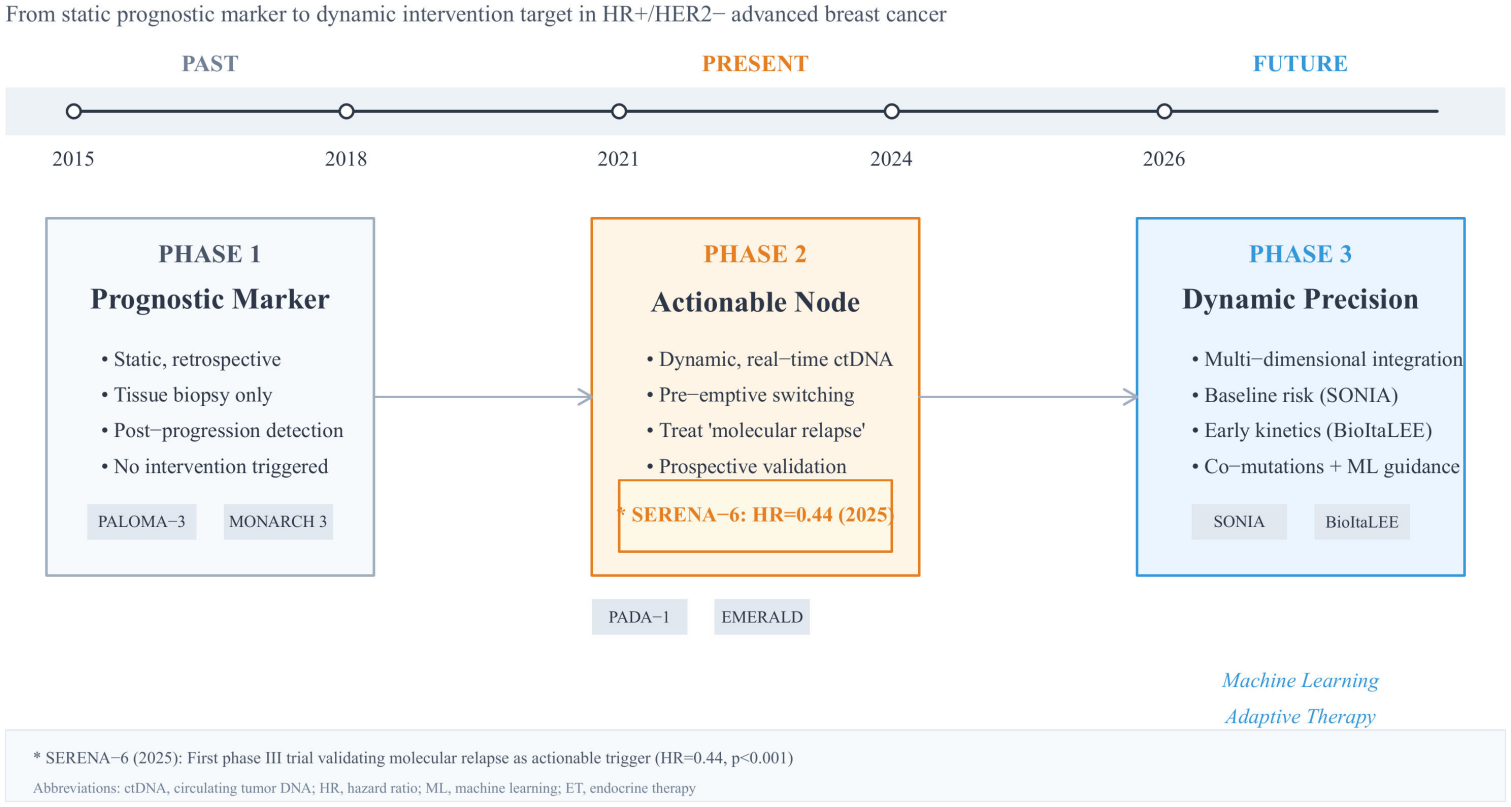
Evolution of ESR1 mutations in clinical practice. Timeline diagram illustrating three phases of ESR1 mutation clinical significance evolution from 2015 to 2026 and beyond: Phase 1 (2015-2021) prognostic marker with static tissue biopsy; Phase 2 (2021-2025) actionable node with dynamic ctDNA monitoring enabling pre-emptive treatment switching; Phase 3 (2026 onward) dynamic precision integrating baseline risk stratification, early ctDNA kinetics, and co-mutation landscapes.

The evolution of *ESR1* mutations in clinical practice (**Figure 2**) represents a paradigm shift from static assessment to dynamic intervention.

#### 4.4.1 Biological rationale for dynamic monitoring

Tumors undergo continuous clonal evolution under therapeutic pressure. *ESR1* mutation incidence increases with subsequent lines, reaching 33% in second-line and up to 40% in later lines (12, 36, 37). Dynamic monitoring captures shifts in the genomic landscape, transforming *ESR1* mutations into dynamic therapeutic decision-making targets.

#### 4.4.2 Prospective validation of dynamic intervention: the SERENA-6 milestone

The phase III SERENA-6 trial provides highest-level evidence. Patients with emergent *ESR1* mutations on first-line AI plus CDK4/6i without radiographic progression were randomized to switch to camizestrant or continue AI. Early switching reduced progression or death risk by 56% (HR = 0.44; P<0.0001), with median PFS nearly doubled (16.0 vs. 9.2 months). This trial formally established “emergent *ESR1* mutations” as an actionable clinical decision node (20).

Complementing this, EMERALD established oral SERD efficacy in *ESR1*-mutant patients after CDK4/6i progression, leading to FDA approval.

#### 4.4.3 Early ctDNA kinetics as efficacy readout

BioItaLEE showed ctDNA clearance at C2D1 reduced progression risk (HR = 0.44), while persistent co-mutations indicated primary resistance (40). The study also found 22.7% of baseline-negative patients developed detectable mutations during treatment. PADA-1 translational analysis confirmed early ctDNA evolution characteristics were significantly associated with poor prognosis (HR for OS = 1.51) (46).

#### 4.4.4 From concept to practice: the PADA-1 paradigm

PADA-1 demonstrated that early switching to fulvestrant upon rising *ESR1* mutations without radiographic progression improved PFS (HR = 0.61; median 11.9 vs. 5.7 months). MONARCH 3 longitudinal analysis reinforced that effective CDK4/6 inhibition may delay *ESR1*-mediated resistance.(Goetz, Hamilton, et al., 2024).

#### 4.4.5 The enduring role of baseline static testing

Baseline static testing identifies high-risk patients prior to therapy initiation, serving as the essential “risk anchor” upon which dynamic monitoring builds.

#### 4.4.6 Current guidelines and practice gaps

Major guidelines, including ESMO and NCCN, have not yet recommended routine ctDNA *ESR1* testing for all patients due to barriers including: (1) the need for prospective clinical utility evidence beyond the trial setting; (2) lack of harmonized testing standards and VAF threshold consensus; (3) absence of agreed-upon monitoring intervals and intervention thresholds; and (4) limited reimbursement and accessibility. These gaps highlight the importance of framing current recommendations within a research context.

### 4.5 The complementary role of post−progression strategies

A comprehensive understanding of the therapeutic landscape requires integrating pre−emptive and post−progression strategies. These approaches are not mutually exclusive but rather constitute a continuum of care:

PADA−1 (Pre−emptive switch): Demonstrated that switching from AI to fulvestrant upon ctDNA−detected *ESR1* mutation emergence—before radiographic progression—significantly prolongs PFS (HR = 0.61). This established the proof−of−concept for ctDNA−guided early intervention.

SERENA−6 (Pre−emptive switch with novel SERD): Validated the pre−emptive switching paradigm using the more potent oral SERD camizestrant, achieving a 56% risk reduction (HR = 0.44). This provides the highest level of evidence for dynamic ctDNA−guided intervention.

postMONARCH and MAINTAIN (Post−progression continuation): Demonstrated that continuing CDK4/6i with a switch in endocrine therapy after radiographic progression provides clinical benefit (*postMONARCH*: HR = 0.73, P = 0.017; *MAINTAIN*: HR = 0.57, 95% CI 0.39−0.95). This establishes a backbone for post−progression management.

EMERALD (Post−progression novel SERD): Showed that in ESR1−mutant patients who have progressed on CDK4/6i, elacestrant significantly improves PFS (HR = 0.55). This provides a targeted option specifically for *ESR1*−mutant tumors.

Collectively, these trials outline a dynamic management paradigm: ctDNA monitoring enables pre−emptive switching to delay progression; after progression, continuing CDK4/6i with endocrine therapy switch or transitioning to oral SERDs (in *ESR1*−mutant cases) offers additional lines of effective therapy. This integrated approach positions ctDNA *ESR1* testing as a continuous surveillance system guiding decisions across the entire treatment trajectory.

## 5 Discussion

### 5.1 Summary of key findings

This review identifies that, in the era of CDK4/6i plus endocrine therapy, ctDNA *ESR1* mutations have evolved through three distinct phases of clinical significance:

Phase 1 (2015-2021, Prognostic Marker): Characterized by static, retrospective analyses using tissue biopsy, with studies such as PALOMA-3 and MONARCH 3 establishing *ESR1* mutations as adverse prognostic factors but without triggering intervention. Pooled evidence from this period demonstrated that baseline *ESR1* mutations are associated with shorter PFS in both first-line and second-line settings, with hazard ratios typically ranging from 2.0 to 3.0. However, these studies also showed that while *ESR1* mutations confer worse prognosis, they do not diminish the relative benefit derived from CDK4/6i combination therapy.

Phase 2 (2021-2025, Actionable Node): Marked by dynamic, real-time ctDNA monitoring enabling pre-emptive treatment switching. The PADA-1 trial provided proof-of-concept that switching from AI to fulvestrant upon ctDNA-detected *ESR1* mutation emergence—before radiographic progression—significantly prolongs PFS (HR = 0.61). This paradigm was definitively validated by the phase III SERENA-6 trial (2025), which demonstrated that pre-emptive switching to camizestrant plus CDK4/6i reduced the risk of disease progression or death by 56% (HR = 0.44, 95% CI 0.31-0.60, P<0.0001), formally establishing “molecular relapse” as an actionable clinical decision node.

Phase 3 (2026 and beyond, Dynamic Precision): Emerging as multidimensional integration of baseline risk stratification, early ctDNA kinetics, co-mutation landscapes, and machine learning-guided adaptive therapy. Key advances include: (1) the SONIA trial demonstrating that high baseline ctDNA tumor fraction (aneuploidy score ≥10%) identifies patients who derive substantial benefit from first-line CDK4/6i (PFS2 HR 0.58), providing an actionable tool for upfront treatment intensification; (44). (2) the BioItaLEE study establishing the “one-month rule,” wherein ctDNA clearance by cycle 2 day 1 correlates with significantly prolonged PFS (HR = 0.44), whereas persistent co-mutations (e.g., *TP53*/MYC) indicate primary resistance; and (3) recognition of mutation subtype heterogeneity, with Y537S exhibiting stronger constitutive activation and poorer prognosis compared to D538G, suggesting a need for subtype-differentiated therapeutic strategies.

Additional key findings include the significant clinical heterogeneity of *ESR1* mutation subtypes, the prognostic value of early ctDNA kinetics independent of conventional imaging, and the importance of co-mutation analysis (particularly *TP53* and *PIK3CA*) in refining risk stratification and treatment selection.

### 5.2 Biological interpretation

Baseline *ESR1* mutations may originate from residual clones resistant to prior AI therapy or *de novo* clones with inherent genomic instability, both gaining growth advantage under CDK4/6i pressure. Mutation subtype matters: Y537S exhibits 2–3× higher transcriptional activity than D538G and is associated with shorter survival, though novel oral SERDs may overcome these differences as shown in EMERALD. ctDNA encodes multidimensional information: high baseline ctDNA fraction identifies patients deriving greatest benefit from first-line CDK4/6i (PFS2 HR = 0.58), while early kinetics reflect drug sensitivity, with failure to clear ctDNA within 1–2 cycles strongly predicting poor outcomes.

### 5.3 Current limitations

This review has several limitations. First, most data derive from retrospective subgroup analyses, with no prospective trials evaluating upfront intensification for baseline *ESR1* mutation carriers. Second, ctDNA detection platform heterogeneity (ddPCR vs. NGS) and non-standardized workflows hinder clinical translation. Third, there is no consensus on optimal VAF thresholds, monitoring intervals, or subtype-specific management approaches. Fourth, the review was not registered in PROSPERO, and despite adherence to PRISMA guidelines, the narrative synthesis design limits the strength of quantitative conclusions. These gaps highlight the need for harmonized prospective research to validate our findings.

### 5.4 Clinical implications

Based on the available evidence, the following implications are proposed as a research−informed framework rather than definitive clinical recommendations:

For patients initiating first−line CDK4/6i therapy, baseline ctDNA *ESR1* testing may be considered in the context of clinical trials or individualized risk−benefit assessment to identify high−risk populations who might be candidates for future intensification strategies. However, routine baseline testing outside of research settings is not yet standard practice.

Regular ctDNA monitoring (every 3−6 months) could enable early detection of emergent mutations, but the optimal frequency and clinical utility outside of trial settings remain to be established.

When an *ESR1* mutation is detected without radiographic progression, evidence from PADA−1 and SERENA−6 supports early switching to SERD−based regimens in the context of the specific trial populations and eligibility criteria. Extrapolation to broader clinical practice requires caution.

Co−mutation analysis (e.g., *TP53, PIK3CA*) may further refine treatment strategies, but this remains an investigational approach pending prospective validation.

The post−progression landscape (Section 4.5) provides a comprehensive framework for sequencing therapy after CDK4/6i progression, integrating evidence from postMONARCH, MAINTAIN, and EMERALD. Recent reviews have comprehensively summarized these strategies (47).

## 6 Future perspectives

### 6.1 Current clinical practice recommendations

Based on the available evidence, we propose the following as a research-informed framework to guide future investigation and individualized clinical decision-making, rather than as definitive practice recommendations. The strength of evidence varies substantially across these suggestions.

For patients initiating first-line CDK4/6i therapy, baseline ctDNA *ESR1* testing may be considered in the context of clinical trials or individualized risk-benefit assessment to identify high-risk populations who might be candidates for future intensification strategies. However, routine baseline testing outside of research settings is not yet standard practice.

Patients with baseline *ESR1* mutations, particularly Y537S, could be considered for more frequent ctDNA monitoring (e.g., every 2–3 months) within structured research protocols, given their higher risk of early progression. The optimal frequency and clinical utility of such monitoring remain to be established.

For patients on active treatment, regular ctDNA monitoring (every 3–6 months) represents a promising approach for early detection of emergent mutations. Evidence from PADA-1 and SERENA-6 supports this strategy in the context of those trials, but extrapolation to routine practice requires caution.

Early ctDNA kinetics (e.g., clearance at cycle 2 day 1) represent an investigational tool for assessing treatment response; failure to clear ctDNA by this timepoint may prompt consideration of early treatment adjustment within a clinical trial framework, but this approach remains experimental pending prospective validation.

When an *ESR1* mutation is detected without radiographic progression, clinical decision−making should integrate mutation subtype, VAF dynamics, and prior treatment history, drawing on evidence from PADA−1 and SERENA−6 that support early switching to SERD−based regimens (21, 38).

### 6.2 Prospective trial design

Future prospective trials should move beyond static baseline assessment toward dynamic risk-response guided models. The most pressing task is to initiate trials enrolling patients with baseline ctDNA *ESR1* mutations to validate whether upfront intensified treatment can overcome their poor prognosis (20). For the baseline high-risk population, the “combination intensification” strategy from SERENA-6 can be adopted, comparing CDK4/6i plus novel oral SERD versus standard CDK4/6i plus AI. Trial designs require further refinement by incorporating early on-treatment ctDNA kinetics as a dynamic decision node. All enrolled patients could first receive a short “run-in” period of standard treatment; those failing to achieve ctDNA molecular response would be randomized to continue or switch to a more potent combination. This “dual precision” model—focusing on high-risk patients while using on-treatment molecular response to guide randomization—holds promise for more accurately identifying patients who require treatment escalation (44).

### 6.3 Exploration of novel therapeutic strategies

Patients with baseline *ESR1* mutations represent an ideal population for testing next-generation endocrine agents, including novel oral SERDs (camizestrant, elacestrant), selective ER degraders, and combinations with CDK4/6i, PI3K/AKT/mTOR pathway inhibitors, or immunotherapy. The clinical validation of elacestrant in EMERALD demonstrates that targeting *ESR1*-mutant tumors with novel oral SERDs translates into meaningful clinical benefit (PFS HR = 0.55 in *ESR1*-mutant population; HR = 0.41 in patients with prior CDK4/6i ≥12 months). For high-transcriptional subtypes such as Y537S, more aggressive upfront combination strategies may be required.

### 6.4 Technology integration and model development

Composite predictive models integrating baseline *ESR1* status, ctDNA kinetics, multi-gene co-mutation profiles, and clinicopathological features should be developed using machine learning methods. Such models have already been validated in large-scale trials: a ctDNA risk model based on the PADA-1 cohort predicted patient survival independently of RECIST criteria (OS HR = 4.10, P<0.001), improving the C-index from 60.3% to 70.0% (P = 0.035) (44). Future efforts should integrate genomic, kinetic, imaging, and clinicopathological dimensions to achieve dynamic prediction of treatment response and early warning of resistance evolution.

### 6.5 In-depth translational research

Living biobanks (patient-derived organoids or xenografts) of *ESR1*-mutant tumors should be established to investigate signaling network dependencies, differential drug sensitivity across mutation subtypes (Y537S vs. D538G), mechanisms by which co-mutations influence resistance, and preclinical validation of novel combination therapies. Single-cell sequencing techniques may further elucidate clonal evolution and heterogeneity.

### 6.6 Strategy extension from advanced to early stage

The value of ctDNA *ESR1* monitoring is progressively extending to early-stage disease. The DARE study has confirmed the feasibility of ctDNA-guided intervention in the adjuvant setting. Future directions include regular ctDNA monitoring to screen for *ESR1* mutations in high-risk early breast cancer patients after adjuvant therapy, proactive endocrine therapy escalation upon molecular relapse without radiographic evidence, and incorporation of “molecular complete response” as a neoadjuvant endpoint. This strategy would advance *ESR1* mutation utility from advanced disease management to curative treatment phases.

## 7 Distinctive aspects of this review

This comprehensive review contributes to the existing literature in three key aspects:

First, while previous reviews have focused on *ESR1* mutations as static prognostic markers, our work synthesizes emerging evidence from nine high−quality studies to demonstrate their evolution into dynamic intervention nodes. The inclusion of the recently published SERENA−6 trial (2025) provides the highest−level evidence that ctDNA−detected emergent ESR1 mutations can serve as actionable triggers for pre−emptive therapeutic switching (HR = 0.44, P<0.0001).

Second, unlike prior narrative reviews, our systematic approach integrates three complementary dimensions: baseline risk stratification (baseline *ESR1* mutations, particularly Y537S, identify high−risk patients), early on−treatment kinetics (ctDNA clearance by cycle 2 day 1 predicts prolonged PFS, HR = 0.44), and clonal evolution tracking (co−mutation analysis with TP53/PIK3CA refines risk prediction).

Third, we propose a practical clinical research framework that translates these evidence streams into testable decision algorithms. This framework distinguishes between “already practice−changing” evidence (SERENA−6, PADA−1, EMERALD) and “urgently needing prospective validation” (upfront intensification for baseline *ESR1* mutation carriers), directly guiding future trial design and clinical implementation.

Collectively, this review provides clinicians and researchers with a comprehensive, evidence−graded roadmap for integrating ctDNA *ESR1* monitoring into precision decision−making for HR+/HER2− advanced breast cancer, while clearly acknowledging the remaining gaps that must be addressed before routine clinical adoption.

## Supplementary material

Supplementary Table 1 Detailed characteristics and technical parameters of included studies.
