# Supplementary material for: Dynamic circulating tumor DNA profiling of ESR1 mutations in HR+/HER2− advanced breast cancer: from prognostic biomarker to clinical decision-making – a comprehensive review
Source: Front Oncol. 2026 Jul 31;16:1881097. doi: 10.3389/fonc.2026.1881097 (PMC13472803; doi:10.3389/fonc.2026.1881097)
Supplement: Supplementary file 1 [file Table1.docx]

**Supplementary Table 1. Detailed Characteristics and Technical Parameters of Included Studies**

| **Study** | **ctDNA Detection Platform** | **VAF Threshold** | **Sampling Timepoints** | **ESR1 Mutation Subtype Analysis** | **HR by Subtype**  **(95% CI)** | **Median Follow‑up** | **Quality Score** |
| --- | --- | --- | --- | --- | --- | --- | --- |
| **MONARCH 3** | NGS (FoundationOne® Liquid CDx) | Not explicitly reported  (standard NGS LOD ~1%) | Baseline, End of Treatment (EOT) | Y537S, D538G, Y537N, Y537C, L536R, L536Q, L536H, P535H, V534E | ITT OS HR (ESR1 mut vs. wt): 0.804 (0.637-1.015); P=0.0664; sVD OS HR: 0.758 (0.558-1.030); P=0.0757 | 8.1 years | Low RoB |
| **PALOMA-3** | ddPCR (PIK3CA) + NGS (ESR1/TP53) | ≥0.1% (ddPCR); ≥1% (NGS) | Baseline, C1D15, progression | D538G, Y537S, Y537N, Y537C, L536Q, L536R, E380Q | OS HR (ESR1 mut vs. wt): 0.81 (0.65-0.99) | 73.5 months | Low RoB |
| **SONIA** | mFast-SeqS (aneuploidy score) | Aneuploidy score ≥10%  (high tumor fraction) | Baseline | Not applicable | PFS2 HR (high vs. low tumor fraction): 0.58 | 47.9-48.1 months | Some concerns |
| **BioItaLEE** | NGS  (39-gene custom panel) | Not explicitly reported (defined as VAF drop to undetectable level) | Baseline, C1D15, C2D1, first radiological assessment (~Week 12), progression | Y537S, D538G, Y537N, Y537C, E380Q, L536Q, L536R, P535H, V534E | PFS HR (clearance at C2D1 vs. no clearance): 0.44; PFS HR (C1D15 clearance): 0.51; PFS HR (baseline mut vs. wt): 0.41; P=0.0001 | 26.9 months (Clin Cancer Res 2026) | High (NOS 9★) |
| **PADA-1** | ddPCR  (custom ESR1 screening assay) | ≥0.1%  (rising mutation defined as VAF increase ≥10‑fold from baseline or absolute VAF ≥1%) | Monthly (first 3 months), then every 2‑3 months | D538G, Y537S, Y537N, Y537C, L536Q, L536R, E380Q, P535H, V534E | PFS HR (early switch vs. continuation): 0.61 (0.43-0.86); P=0.004 | 26.0 months | Low RoB |
| **SERENA‑6** | High-sensitivity NGS (Guardant360® CDx) | ≥0.2%  (central laboratory) | Every 2‑3 months (screening); baseline, C1D1, C2D1, then every 8 weeks | Y537S, D538G, Y537N, Y537C, L536Q, L536R, E380Q, P535H, V534E | PFS HR (camizestrant vs. AI continuation): 0.44 (0.31-0.60); P<0.0001 | 11.0 months (NEJM 2025) | Low RoB |
| **EMERALD** | ctDNA‑based ESR1 detection (Guardant360®) | Not explicitly reported  (central laboratory) | Baseline, C1D1, progression | Y537S/N, D538G | ESR1 mut (all) PFS HR: 0.55 (0.39-0.77); Prior CDK4/6i ≥12m: 0.41 (0.26-0.63); Y537S/N: 0.25 (0.13-0.47); D538G: 0.38 (0.21-0.67) | 13.4 months | Low RoB |
| **postMONARCH** | NGS (FoundationOne® Liquid CDx) | Not explicitly reported  (standard NGS LOD ~1%) | Baseline, C1D1, EOT, progression | Y537S, D538G, Y537N, Y537C, L536Q, L536R, E380Q, P535H, V534E | ITT PFS HR: 0.73 (0.57-0.95); P=0.017; BICR PFS HR: 0.55 (0.39-0.77); ESR1 mut HR: 0.79; ESR1 wt HR: 0.78 | 11.0 months (per primary analysis) | Low RoB |
| **MAINTAIN** | NGS (Guardant360®) | Not explicitly reported (standard NGS LOD ~1%) | Baseline, progression | Y537S, D538G, Y537N, Y537C, L536Q, L536R, E380Q | PFS HR (ribociclib + ET vs. placebo + ET): 0.57 (0.39-0.95) | 11.8 months | Low RoB |
